# Polyphenol Composition, Antioxidant Properties, and Health Benefits of Moroccan-Cultivated Raspberries, Blackberries, and Blueberries: A Comprehensive Review

**DOI:** 10.3390/foods15081356

**Published:** 2026-04-13

**Authors:** Abderrahim Alahyane, Samira El Qarnifa, Abdoussadeq Ouamnina, Bouchra El Hayany, Imane El ateri, Abdelaziz Mounir, Hassan Alahyane, Mourad Ouhammou, Mohamed Abderrazik

**Affiliations:** 1 Laboratory of Water Sciences, Microbial Biotechnologies, and Natural Resource Sustainability, Faculty of Sciences Semlalia, Cadi Ayyad University, Marrakesh 40000, Morocco; 2 Higher Institute of Nursing Professions and Health Techniques of Guelmim (ISPITSG), Guelmin 81000, Morocco; 3 Laboratory of Agro-Food, Biotechnologies and Valorization of Plant Bioresources (AGROBIOVAL), Faculty of Science Semlalia, Cadi Ayyad University, Marrakesh 40000, Morocco; 4 Laboratory of Excellence in Agrobiotechnology and Bioengineering, AgroBiotech Center, CNRST-Accredited Research Unit (URL05-CNRST), Abiotic Stress Physiology Team, Faculty of Sciences Semlalia, Cadi Ayyad University, Marrakesh 40000, Morocco; ouamnina@gmail.com (A.O.);; 5 Higher Institute of Nursing Professions and Health Techniques, Essaouira-Marrakesh 44000, Morocco; 6 Higher Institute of Nursing Professions and Health Techniques, Beni Mellal 23000, Morocco; 7 Higher Institute of Nursing Professions and Health Techniques (ISPITS), Marrakesh 40000, Morocco; ouhamoumourad@hotmail.com

**Keywords:** berries, phenolic compounds, antioxidant, health benefits, Morocco, functional foods

## Abstract

Despite Morocco’s emergence as the world’s fourth-largest berry exporter, no comprehensive review has evaluated the polyphenol composition, antioxidant properties, and health benefits of raspberries (*Rubus idaeus*), blackberries (*Rubus fruticosus*), and blueberries (*Vaccinium corymbosum*) specifically within the Moroccan cultivation context. This narrative review synthesized evidence from phytochemical analyses, in vitro and in vivo studies, randomized controlled trials (RCTs), meta-analyses, and epidemiological data sourced from PubMed, Scopus, and Web of Science. Blackberries exhibited the highest total polyphenol content (149 μmol GAE/L) and antioxidant capacity, driven primarily by anthocyanin concentration and diversity. Antioxidant mechanisms included free radical scavenging, transition metal chelation, and upregulation of endogenous antioxidant enzymes. Pooled RCT data demonstrated that regular consumption (150–300 g/day) significantly reduced systolic blood pressure (−2.72 mmHg), LDL cholesterol (−0.21 mmol/L), and fasting glucose (−2.70 mg/dL). Additional benefits included neuroprotection via blood-brain barrier crossing and brain-derived neurotrophic factor (BDNF) elevation, prebiotic modulation of *Bifidobacterium*, *Lactobacillus*, and *Akkermansia* populations, and anti-cancer activity via nuclear factor-kappa B (NF-κB) and mitogen-activated protein kinase (MAPK) inhibition. Processing significantly affected bioactive retention: freezing preserved phenolic compounds effectively, while conventional drying reduced anthocyanin content by up to 49%. These findings support the integration of Moroccan-cultivated berries—particularly from the Gharb, Loukkos, and Souss-Massa regions—into evidence-based dietary and functional food strategies. Priority research gaps include bioavailability assessment, dose-response characterization, and cultivar-specific phytochemical profiling under Moroccan agro-climatic conditions.

## 1. Introduction

Berries are increasingly recognized as important components of a healthy diet due to their rich content of bioactive compounds [1]. These fruits contain high levels of flavonoids, phenolics, anthocyanins, vitamins, and minerals that exhibit potent antioxidant, anticancer, anti-inflammatory, and neuroprotective properties [2,3]. Numerous studies have demonstrated the potential of berries in preventing chronic diseases such as cancer, cardiovascular disorders, diabetes, and obesity, with health benefits attributed to the synergistic effects of their phytochemicals [4,5].

Morocco’s berry cultivation has seen significant growth in recent years. Blueberry production expanded from experimental trials in the early 2000s, with large-scale commercial production taking off in 2004, reaching 35,100 tons by 2020, a remarkable 19-fold increase from 2005 output [6]. In the 2023/2024 season, Morocco exported approximately 67,300 tons of fresh blueberries, representing a 25% increase from the previous season [6]. Research on new cultivars, including varieties developed through breeding programs by major companies, groups, and cooperatives [6], shows potential for adaptation to Moroccan conditions.

Berries are particularly rich in phenolic compounds, especially anthocyanins, which have been associated with antioxidant properties and may help reduce oxidative stress [7]. Phenolic compounds exhibit anti-inflammatory effects by inhibiting enzymes involved in eicosanoid production and molecular signaling pathways activated by oxidative stress [8]. However, the low bioavailability of phenolic compounds compared to endogenous antioxidants has led researchers to question their role as mere antioxidants [9].

Researchers have developed efficient and environmentally friendly extraction methods to recover antioxidant compounds from berries, aiming to reduce production costs, limit pollution, and preserve bioactive compounds [10]. Advanced techniques such as ultrasound-assisted extraction, enzyme-assisted extraction, and microwave-assisted extraction improve the release, diffusion, and dissolution of intracellular antioxidants [11,12].

In recent years, berries have gained increasing research attention owing to their expanding production, recognized health benefits, medicinal properties, and minimal toxicity [13]. Existing reviews have exanimated various aspects of berries including botanical characteristics, nutritional and antioxidant properties [14], pharmacological activities, and potential applications in the food industry [13,15]. However, although berries produced in Morocco are increasingly recognized for their nutritional and antioxidant potential, the available literature remains fragmented regarding the characterization, extraction methods, and health-related applications of their bioactive compounds.

Further research is needed to determine optimal therapeutic doses and mechanisms behind the health benefits of berry phenolic compounds [12]. Despite the growing interest in berry consumption and proven health advantages, more clinical trials are needed to fully understand the bioavailability and therapeutic potential of berry compounds [16].This comprehensive narrative review aims to comprehensively evaluate the polyphenol composition, antioxidant properties, and health benefits of Moroccan-cultivated raspberries, blackberries, and blueberries.

## 2. Botanical and Agricultural Overview

### 2.1. Taxonomy and Botanical Features

**Figure 1:**
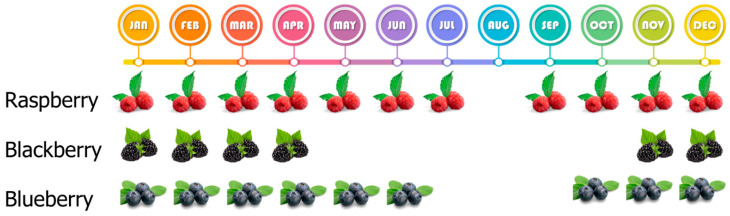
Seasonal production calendar of selected berries in Morocco (Created by A. Alahyane—2025).

Berry taxonomy provides a systematic classification of commercially important berry fruits within the plant kingdom. The primary berries of commercial importance, raspberry (*Rubus idaeus*), blackberry (*Rubus fruticosus*), and blueberry (*Vaccinium corymbosum*), belong to two distinct plant families: *Rosaceae* and *Ericaceae* (Figure 1). The *Rosaceae* family includes both raspberry and blackberry within the genus *Rubus*, while blueberries belong to the *Vaccinium* genus within the *Ericaceae* family (Table 1). In Morocco, the total surface area dedicated to berry cultivation reaches 5700 hectares, distributed among blueberry (1200 ha), raspberry (1100 ha), and blackberry (less than 20 ha). Raspberry production was launched in 2005 and blueberry in 2007, driven by European companies relocating to the region alongside local agricultural enterprises. By 2017, total production exceeded 173,000 tonnes, with blueberry reaching 12,000 tonnes and raspberry 11,000 tonnes. The sector comprises approximately 20 farms for raspberry and 12 for blueberry, with facilities ranging from 1–5 ha to over 60 ha. Regarding market outlets, 90% of raspberry volumes and 100% of blueberry volumes are destined for export, primarily to the European market (94% of exports). Fresh berry exports exceeded 38,000 tonnes in 2016–2017, with raspberry exports surpassing 10,000 tonnes. The sector objective for 2020 was set at 8200 ha and 360,000 tonnes per year, of which 80,000 tonnes earmarked for export [17].

#### 2.1.1. Rubus Genus Overview

The genus *Rubus*, belonging to the Rosaceae family, contains between 600 and 1000 species distributed across 12 subgenera, thriving in ecosystems from tropical to subarctic regions. Within the *Rubus* genus, the subgenera *Idaeobatus* (raspberries) and *Rubus* (blackberries) are most economically important, with cultivated varieties produced in more than 43 countries worldwide [18].

A distinctive feature of the *Rubus* genus is its varied ploidy levels. Raspberries are predominantly diploid (2*n* = 2x = 14), while blackberries exhibit a range from diploid to dodecaploid (2*n* = 2x = 14 to 2*n* = 12x = 84) [19].

Red raspberries are perennial plants with biennial canes that produce fruit in their second year. The fruits are aggregate drupelets that develop from multiple ovaries, creating the characteristic hollow structure when harvested [20]. Blackberries differ from raspberries in that the receptacle (torus) remains attached to the fruit at harvest, resulting in a more solid structure.

#### 2.1.2. Vaccinum Genus Overview

Blueberries belong to the genus *Vaccinium* within the *Ericaceae* family, which includes approximately 450 species. The northern highbush blueberry (*Vaccinium corymbosum*) is the most widely cultivated species commercially.

Blueberry plants are woody perennial shrubs with distinctive bell-shaped flowers and glaucous blue fruits. Unlike *Rubus* species, blueberries are diploid (2*n* = 2x = 24) with a relatively compact genome [19]. The fruits are true berries containing multiple small seeds, differing from the aggregate structure of raspberries and blackberries. Recent genomic resources have expedited breeding of improved cultivars with enhanced traits including flavor, yield, and disease resistance [21].

### 2.2. Cultivation in Morocco

#### 2.2.1. Geographic Regions

Berry cultivation in Morocco, particularly of raspberries, blackberries, and blueberries, has been gaining traction in specific geographic regions that offer suitable microclimates [22]. In northern and northwestern Morocco, berries are sourced from different locations such as Moulay Bousselham, Larache, reflecting their geographic distribution within the country [23].

The Loukkos region in northwestern Morocco has emerged as an important area for berry production. In Morocco, raspberry production is mainly concentrated in the northern part of the country but has expanded further south, particularly in the semi-arid Souss-Massa region [23]. This coastal region benefits from the moderating influence of the Atlantic Ocean, creating milder temperatures and higher humidity levels [24]. The area’s sandy loam soils provide good drainage, essential for preventing root diseases in *Rubus* species [25].

In southern Morocco, the Souss-Massa region has begun experimental cultivation of berries under protected agriculture systems like greenhouses [26]. This controlled environment approach helps mitigate the region’s hotter climate while taking advantage of abundant sunshine [27]. Blueberry cultivation has shown particular promise in these settings through appropriate soil amendments and fertigation techniques [28].

Agricultural practices for berry cultivation in Morocco include efficient drip irrigation systems to conserve water mulching to maintain soil moisture [29], and focus on early-season production to target export windows to many markets [30].

**Figure 2:**
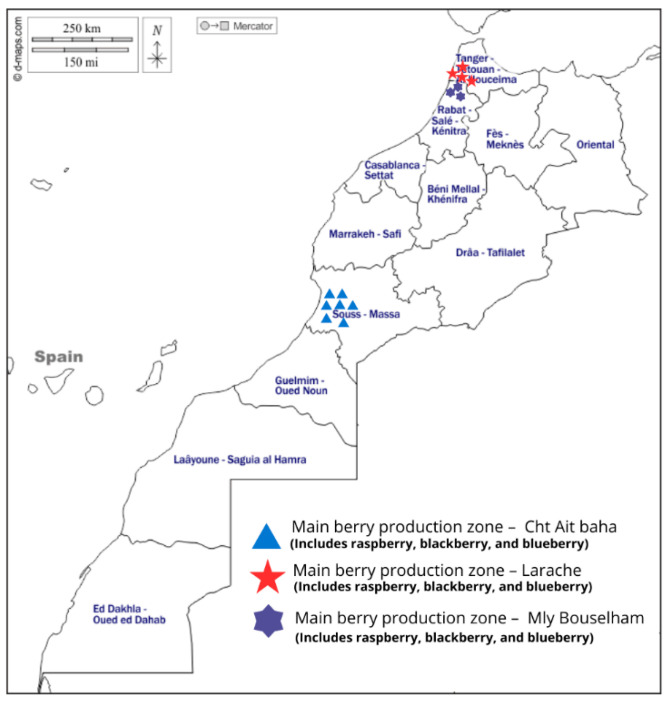
Regions involved in the cultivation or experimental trials of raspberry, blackberry, and blueberry, in Morocco (Created by A. Alahyane—2025).

Despite these developments, berry cultivation in Morocco (Figure 2) remains relatively limited compared to traditional crops. Challenges include specialized knowledge requirements, high initial investment costs, limited availability of adapted cultivars, and competition from established European producers [22]. However, growing global demand for berries and Morocco’s strategic geographic position suggest potential for continued growth [31].

Recent studies in Morocco have explored agricultural practices for berry cultivation, focusing on irrigation, soil composition, and climate adaptation [26]. Pulsed drip irrigation improved blueberry yield, fruit size, and water efficiency compared to continuous irrigation in sandy soils [32,33].

#### 2.2.2. Harvesting and Post-Harvest Handling

##### Harvesting Methods and Timing

**Figure 3:**
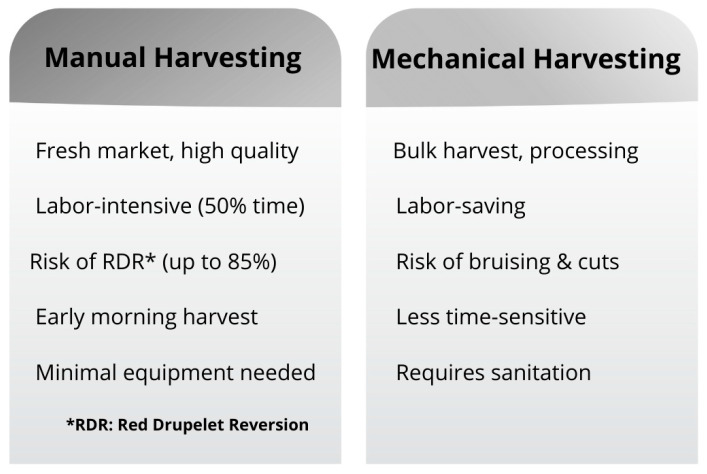
Comparative manual vs. mechanical harvesting.

Berry harvesting methods can categorized into manual and mechanical approaches (Figure 3). Hand harvesting remains predominant for fresh market blackberries and raspberries to maintain quality, though it is labor-intensive and costly, accounting for up to 50% of worker hours [34].

Berries are typically harvested in early morning hours during dry weather, with only healthy, undamaged fruits selected [35]. For blackberries, coloring is most commonly used to determine harvest readiness [36].

Mechanical harvesting has emerged as an alternative to address labor shortages. For blueberries, over-the-row (OTR) harvesters use plastic beating bars mounted on rotary shaking drums [37]. However, mechanical harvesting can increase bruising and cuts, potentially shortening shelf life [38].

Fruit ripeness at harvest significantly influences post-harvest disease development. Botrytis fruit rot is more common in early harvests, while anthracnose and alternaria occur more frequently in late harvests [38]. Shorter harvest intervals may reduce fruit rot risk, particularly for cultivars lacking high firmness [39].

##### Post-Harvest Handling and Storage

**Figure 4:**
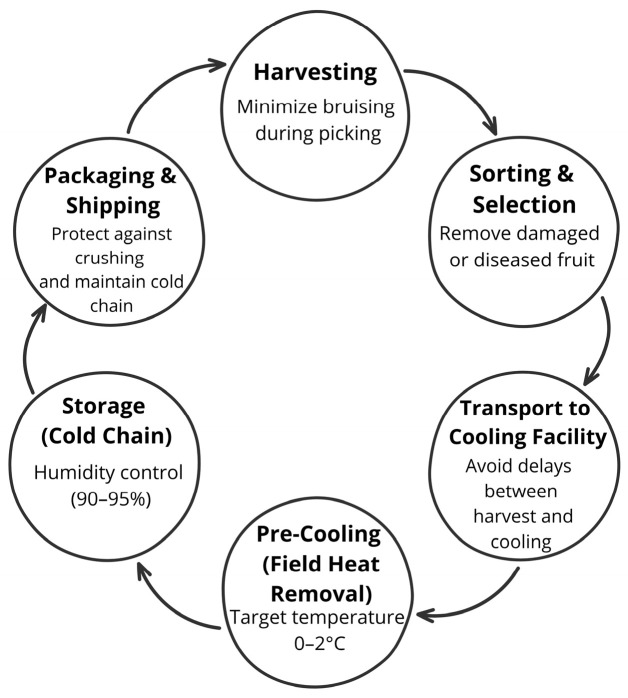
Post-harvest handling workflow for berry fruits.

After harvest, berries require immediate attention due to their highly perishable nature (Figure 4). Blueberries should be promptly sorted and placed in cold storage at 0 °C to reduce respiration and extend shelf life [40]. Controlled atmosphere (CA) storage and modified atmosphere packaging (MAP) can extend shelf life by reducing oxygen levels and increasing carbon dioxide concentrations [41]. Freezing is the most common preservation method for berries, maintaining nutritional value when properly executed [42]. The frozen product is typically sold in bulk packages for use in jams, preserves, or juices [43].

As a leading exporter to the European Union, Morocco faces a critical logistical challenge: the “food mile” versus bioactivity trade off. While Moroccan berries benefit from optimized biosynthesis in the field, long distance transport and the complexities of cold chain management may lead to significant degradation of sensitive anthocyanins [44]. A critical research priority is to assess the stability of the phenolic matrix from the farm gate in Souss Massa or Gharb to the European consumer’s table.

## 3. Polyphenol Composition of Blackberries, Blueberries and Raspberries

### 3.1. Overview of Polyphenol Classes

Phenolic compounds are naturally occurring compounds characterized by aromatic rings bearing hydroxyl groups, contributing significantly to the health-promoting properties of berries [45]. These secondary metabolites can be classified into phenolic acids, flavonoids (including anthocyanins, flavonols, and flavanols) and tannins [46].

The primary phenolic compounds in berries include anthocyanins, flavonols, hydroxycinnamic acid, ellagic acid, ellagic tannins, and proanthocyanins, contributing to bioactive properties and antioxidant capacity [14]. Berries are particularly rich in phenolic acids, flavonoids, tannins, stilbenes, and vitamins C and E, collectively contributing to cancer risk reduction and anti-inflammatory [47]. The antioxidant activity of these phenolic compounds helps protect cells from free radical damage [48]. Phenolic compounds also influence berry quality and flavor, contributing to color, astringency, and bitterness [49].

Polyphenol composition and concentration are highly variable and influenced by a complex interplay of factors, including genotype, geographic origin, environmental conditions, agronomic practices, ripeness stage, harvest timing, and post-harvest handling (in Table 2 and Table 3) [50].

**Table 2:** Total phenols content of raspberries, blackberries, and blueberries.

Matrix Source	Total Phenols Content Detected		Detection Methods and Extraction Conditions	Spectrophotometric Analysis	Geographical Specificity	Plant Part Used	References
**Raspberry**	Total phenols content (mg GAE/100 g FW)	-Red raspberry: 265.83 ± 0.95	-Folin-Ciocalteu method -Distilled water as an extractant	Spectrophotometer at 725 nm	-Larache (Morocco)	Entire fruit	[51]
		-Black raspberry: 305.90 ± 0.00			-Rabat (Morroco)		
**Raspberry**	Total phenols content (mg GAE/100 g FW)	-Red raspberry: 256 ± 0.09 279 ± 0.09	-Folin-Ciocalteu method -Distilled water as an extractant	Spectrophotometer at 725 nm	-Larache (Morocco) -Rabat (Morroco)	Entire fruit	[51]
		-Red raspberry: 286.20 ± 0.10 270.00 ± 0.90 301.70 ± 0.10 -Black raspberry: 307.10 ± 0.10					
**Raspberry**	Total phenols content (mg GAE/100 g FW)	108.23 ± 6.62 and 269.90 ± 6.88	-Foline Ciocalteu method -80% methanol as extractant	-Spectrophotometer at 750 nm	Xiaohe Mountain in Hangzhou, Zhejiang (China)	Entire Fruit	[52]
**Raspberry**	Total phenols content (mg GAE/100 g FW)	175.90–1351.59	-Folin-Ciocalteu method Methanol 80% as extractant	-Spectrophotometer at 750 nm	Brzezna, (Southern Poland)	Entire Fruit	[53]
**Blackberry**		263.09–629.09					
**Raspberry**	Total phenols content (mg GAE/100 g FW)	357.83 ± 7.06	-Methanol/water (50:50, *v*/*v*) as extractant -Folin–Ciocalteu method	-Spectrophotometer at 750 nm	Minas Gerais (Brazil)	Entire Fruit	[53]
**Blackberry**		850.52 ± 4.77					
**Blackberry**	Total phenols content (mg GAE/g DW)	-37.4 ± 3.10 (Had Gharbia Location) -50.2 ± 2.50 (Ghezaoua Location) -63.1 ± 0.20 (Beni Messaouar Location)	-Folin–Ciocalteu method -n-hexane homogenized with ethanol methanol water (80:20 *v*/*v*)	-Spectrophotometer at 755 nm	Tangier–Tetouan–Al Hoceima region	Entire fruit	[54]
**Blueberry**	-Total phenols content (mg GAE/100 g FW)	-206.2 ± 3.9 and 460.4 ± 3.3	-Folin-Ciocalteu method -Extracted with 80% acetone until colorless.	Spectrophotometer at 760 nm	Chine	Entire fruit	[55]
**Blueberry**	-Total phenols content (mg GAE/100 g of DW)	6.0 ± 0.1	Folin-Ciocalteu method -Acidified methanol (which contained HCl 0.1% *v*/*v*) as extractant	Spectrophotometer at 765 nm Gallic acid as a standard	San José, Los Ríos Region (Chile)	Entire fruit	[56]
**Blueberry**	-Total phenols content (mg GAE/100 g of FW)	2.63 ± 0.16	-Folin-Ciocalteu method -Methanol and a drop of HCl (0.1% hydrochloric acid) as extractant	Spectrophotometer at 750 nm	Kenitra region	Entire fruit	[57]
**Blueberry**	-Total phenols content (mg GAE/100 g of DW)	Flesh: 2.00 ± 0.01	-Folin-Ciocalteu method Distilled water as extractant	Spectrophotometer at 765 nm	Mountains of Al-Jabal Al-Akhdar (Oman)	Flesh and seeds	[58]
		Seeds: 0.06 ± 0.00					
**Blueberry**	-Total phenols content (mg of GAE/100 g of DW)	14.89 ± 0.04 and 19.15± 0.17	-Folin-Ciocalteu method -Absolute methanol as extractant	Spectrophotometer at 700 nm	Cotija (Mexico)	Entire fruit	[59]

Abbreviations: GAE: gallic acid equivalents; DW: Dry weight; FW: Fresh weight.

**Table 3:** Phenolic composition of raspberries, blackberries, and blueberries.

Matrix Source	Mobile Phase (Gradient)/Flow Rate (ml·min^−1^)	Column Type and Temperature (°C)	Wavelength (λ) nm	Major Phenolic Compounds Identified	Geographical Specificity	Plant Part Used	References
Raspberry	purified water (A), methanol (B) and purified water and glacial acetic acid 96:4 (*v*/*v*) (C), 0.8	Zorbax Eclipse Plus C18 (250 mm × 4.6 mm i.d. × 5 µm), at 25 °C	280, 303, 330 and 360 nm	-Gallic acid: 1.23 mg/100 g DW -Syringic acid: 2.76 mg/100 g DW -Ferulic acid: 0.27 mg/100 g DW -Quercetin: 1.52 mg/100 g DW	Sibiu County, (Romania)	Entire Fruit	[60]
	A (H_2_O), B (CH_3_OH) and C (H_2_O: CH_3_COOH = 96:4), 0.8	Zorbax Eclipse Plus C18 (250 mm × 4.6 mm i.d. × 5 µm), at 25 °C.	280, 303, 330 and 360 nm	-Gallic acid: 0.89 mg/100 g DW -Syringic acid: 2.76 mg/100 g DW -Ferulic acid: 0.27 mg/100 g DW -Quercetin: 1.70 mg/100 g DW	Transylvania region (Romania)	Entire Fruit	[61]
Blackberry	A (methanol–acetic acid–water, 10:2:88) and B Methanol–acetic acid–water, 90:2:8), 1	HPLC (Agilent Technologies, Santa Clara, CA, USA) (250 mm × 4.6 mm with a 4-μm) ODS column	254 and 280 nm	-Gallic acid: 2.19 and 9.42 mg/100 g -Caffeic acid: 1.15 and 12.89 mg/100 g -p-Coumaric acid:0.39 and 1.26 mg/100 g -o-Coumaric acid:0.01 and 0.06 mg/100 g -Ferulic acid: 0.38 and 2.47 mg/100 g -Ellagic acid:10.61 mg/100 g and 51.50 mg/100 g -Catechin: 111.59 and 438.97 mg/100 g -Rutin: 0.97 and 11.83 mg/100 g -Quercetin: 0.21 and 0.53 mg/100 g	Malatya (Turkey)	Entire Fruit	[62]
	A (acetonitrile) and B (1% formic acid),1	Luna C18(2) RP column, 5 µm, 250 × 4.6 mm, with a C18 guard column, 4 × 30 mm; UV detection at 280, 320, and 360 nm.	280, 320 and 360 nm	-Protocatechuic acid: 3.36 and 27.93 mg/g of pomace extract -Gallic acid: 9.57 and 10.46 mg/g of pomace extract -Catechin: 1.63 and 1.87 mg/g of pomace extract -Epicatechin: 1.17 and 1.47 mg/g of pomace extract -Vanillic acid: 9.55 and 13.09 mg/g of pomace extract -Caffeic acid: 0.20 and 0.46 mg/g of pomace extract -Syringic acid: 5.60 and 6.42 mg/g of pomace extract -Ellagic acid: 0.41 and 0.60 mg/g of pomace extract -Coumaric acid: 0.04 and 0.12 mg/g of pomace extract -Ferulic acid: 0.09 and 0.14 mg/g of pomace extract -Rutin: 0.56 and 0.77 mg/g of pomace extract -Myricetin: 0.10 and 0.12 mg/g of pomace extract -Quercetin: 0.02 and 0.04 mg/g of pomace extract	Lipolist (Serbia)	Pomaces obtained after juice separation	[63]
Blueberry	A: water including 0.1% trifluoroacetic acid, and B: methanol	HPLC-PAD	280, 149, 324, 520, and 534 nm	-p-Coumaric: 80.7 and 225.2 (μg/100 g FW) -Caffeic acid: 0.4 and 35.6 (μg/100 g FW) -Ferulic acid: 26.7 and 185.5 (μg/100 g FW) -Salicylic acid: 21.9 and 172.6 (μg/100 g FW) -Quercetin: 202.8 and 266.7 (μg/100 g FW) (+)-Catechin: 180.1 and 338.8 (μg/100 g FW)	Chine	Entire fruit	[55]
	A (H_2_O), B (CH_3_OH) and C (H_2_O: CH_3_COOH = 96:4), 0.8	Zorbax Eclipse Plus C18 (250 mm × 4.6 mm i.d. × 5 µm), at 25 °C.	280, 303, 330 and 360 nm	-Gallic acid: 16.86 mg/100 g DW -Syringic acid: 9.48 mg/100 g DW -Ferulic acid: 39.59 mg/100 g DW -Quercetin: 0.44 mg/100 g DW -Rutin: 37.51 mg/100 g DW -Caffeic acid: 15.05 mg/100 g DW (+)-Catechin: 11.53 mg/100 g DW -Chlorogenic acid: 93.23 mg/100 g DW	Transylvania region (Romania)	Entire Fruit	[61]

Abbreviations: DW: Dry weight; FW: Fresh weight.

### 3.2. Flavonoids in Raspberries, Blueberries and Blackberries

Flavonoids represent the largest and most diverse class of polyphenolic compounds in berries, with anthocyanins being the most abundant and visually distinctive subclass [64]. Anthocyanins are glycosylated derivatives of anthocyanidins responsible for the red, purple, and blue pigmentation in berries. The six most common anthocyanidins found in nature are cyanidin, delphinidin, pelargonidin, peonidin, petunidin, and malvidin [65].

The anthocyanin composition varies distinctly among berry types. Cyanidin-based anthocyanins predominate in blackberries and raspberries, while blueberries contain a more diverse anthocyanin profile including malvidin, delphinidin, petunidin, cyanidin, and peonidin glycosides [66]. Blackberries contain total anthocyanins ranging from 2.91 to 8.78 mg cyanidin-3-O-glucoside equivalent/g DW across cultivars, with cyanidin-3-O-glucoside as the predominant form, representing approximately 92% of total anthocyanins [67]. Anthocyanins constitute the dominant polyphenol subclass in blackberries, exceeding phenolic acids and flavan-3-ols in quantity. In addition, anthocyanin stability is highly influenced by storage conditions. Studies on blueberry extracts have demonstrated that degradation is significantly accelerated under light exposure compared to cold or dark storage, following zero-order kinetics in illuminated conditions [68]. Consequently, berries subjected to transportation and retail display may contain substantially reduced levels of flavonoids compared to freshly harvested fruits.

Flavonols, including quercetin, kaempferol, and myricetin glycosides, are present in all berry types but in lower concentrations than anthocyanins [69]. These compounds contribute to antioxidant capacity and health benefits despite lower abundance. Flavanols, particularly catechins and proanthocyanidins (condensed tannins) are also present in berries. Seeds and leaves contain higher concentrations than fruits [70].

### 3.3. Phenolic Acids in Raspberries, Blueberries and Blackberries

Phenolic acids appear in berries as hydroxycinnamic acids and hydroxybenzoic acid derivatives, contributing significantly to quality, flavor, astringency and health-promoting properties [71].

Blueberries are particularly notable for their high content of chlorogenic acid content, identified as the most abundant single phytochemical with demonstrated benefits for lipid metabolism [4]. Blueberry phenolic acids consist mainly of hydroxycinnamic acids, including caffeic and p-coumaric acids, alongside flavonols and proanthocyanidins as other key polyphenol subclasses. Total polyphenol content ranges from 48 to 304 mg GAE/100 g of fresh fruit weight, with anthocyanins (malvidin, delphinidin, petunidin, cyanidin, and peonidin) accounting for up to 60% of total polyphenolics in ripe fruit [66].

Blackberries and raspberries are particularly rich in ellagic acid and ellagitannins [72,73]. These compounds contribute significantly to antioxidant capacity and ability to reduce oxidative stress and inflammation.

Gallic acid and derivatives are the main phenolic acids in berry seeds, with blackberry seeds containing highest amounts (13 mg GAE/g defatted seed meals), followed by raspberry (7 mg GAE/g) and blueberry (2 mg GAE/g) [70].

The comparative analysis of phenolic compounds in berries (blackberry, blueberry, and raspberry) highlights a strong variability related to geographical origin and extraction conditions. For blackberries, total polyphenol contents observed in the Tangier–Tetouan–Al Hoceima region range from 37.4 to 63.1 mg GAE/g DW [54]. These values appear overall higher than those reported in Brazil (850.52 mg GAE/100 g FW) [53] and in Poland (263.09–629.09 mg GAE/100 g DW) [74]. Regarding blueberries, Moroccan samples from the Kenitra region show a very low content (2.63 mg GAE/100 g FW) [57] compared to values reported in China (206.2 to 460.4 mg GAE/100 g FW) [55] or Mexico (14.89 to 19.15 mg GAE/100 g DW) [59]. For raspberries, total polyphenol contents in Morocco range between 256 and 305.90 mg GAE/100 g FW depending on location (Larache and Rabat) [51]. These values are comparable to those reported in China (108.23 to 269.90 mg GAE/100 g FW) [52], but lower than some values observed in Brazil (357.83 mg GAE/100 g FW) [75], and especially in Poland where a very high variability is noted (175.90 to 1351.59 mg GAE/100 g FW) [74].

Overall, these variations confirm that the phenolic composition of berries is strongly influenced by environmental factors, geographical origin, genotype, as well as cultivation conditions and agricultural practices, thereby affecting their antioxidant potential and nutritional value [76,77,78,79,80,81].

### 3.4. Tannins and Stilbens in Blackberries, Raspberries and Blueberries

Among the three berries, blackberries display a significantly higher concentration of tannins [82]. Tannins, classified into hydrolyzable tannins and condensed tannins (proanthocyanidins), are significant polyphenolic compounds contributing to astringency and antioxidant properties [14]. Blueberries contain both hydrolyzable and condensed tannins. Proanthocyanidins are particularly abundant in blueberries, with B-type procyanidin dimers identified as dominant compounds in blueberry seeds [70].

Stilbenes, such as resveratrol and pterostilbene, are found in berries in smaller quantities than tannins and flavonoids and varies significantly by species. Blueberries contain stilbenoids alongside other phenolic compounds [83].

### 3.5. Comparative Polyphenol Content Among Blackberries, Raspberries and Blueberries

Blackberries generally contain the highest total phenolic content, followed by raspberries and blueberries [84,85]. One study reported total phenolic content in blackberries at approximately 149 μmol GAE/L, compared to 116 μmol GAE/L in raspberries and 112 μmol GAE/L in blueberries [85]. The other study found blackberries contained around 10.46 mg GAE/g dry weight of total polyphenols, substantially higher than blueberry varieties (4.57–5.62 mg GAE/g) [84].

The anthocyanin profile varies markedly among berry species. Blackberries exhibit substantially higher anthocyanin content (approximately 1673 mg/100 g fresh weight) compared to raspberries (314 mg/100 g), with cyanidin-3-O-glucoside being the predominant compound in blackberries [86]. In contrast, blueberries are characterized by a more diverse anthocyanin composition, including glycosides of malvidin, delphinidin, petunidin, cyanidin, and peonidin, which together can account for up to 60% of total polyphenols [87]. Furthermore, while blueberries are particularly rich in chlorogenic acid, blackberries and raspberries are distinguished by their higher content of ellagitannins, highlighting clear compositional differences among these berry types.

### 3.6. Influence of Cultivation Conditions on Polyphenol Content

Reported polyphenol contents vary widely even for the same berry species (Table 2). For instance, total phenolic content in raspberries ranges from 48 to 304 mg GAE/100 g FW, and similar variability exists for blueberries and blackberries. This heterogeneity stems from differences in cultivar, maturity stage, growing conditions, extraction methods, and analytical protocols (e.g., Folin–Ciocalteu conditions, reference standards). Such variability complicates cross study comparisons and makes it difficult to establish reliable dose–response relationships. A critical view acknowledges that absolute values from individual studies should be interpreted as indicative rather than definitive.

Environmental factors such as light and temperature significantly affect phenolic compound composition. In blueberries, soil management systems positively influence synthesis and accumulation of flavonols and flavan-3-ols in berries and leaves, enhancing anthocyanin accumulation [88,89]. Additionally, climatic conditions such as water stress increase polyphenol levels in blueberries as an adaptive response [76]. Consequently, under Morocco’s semi-arid climate, cultivated berry species tend to exhibit higher concentrations of bioactive compounds compared to those grown in more temperate regions.

Ripening stage critically affects polyphenol content. In blueberries, average phenolic content increases from 196.49 mg/100 g in unripe fruit to 255.12 mg/100 g in ripe fruit [47]. During development, proanthocyanidins and flavonols are major compounds in early stages, while anthocyanin accumulation begins at ripening onset [90].

In continuation of the synthesis of phenolic compounds in fruits, particularly berries, cultivation conditions emerge as a key factor influencing the biosynthetic pathways of phenolic compounds. Understanding plant–environment interactions therefore represents a strategic lever for guiding agricultural practices toward the production of berries with high functional value.

Several studies indicate that organic farming may promote a higher accumulation of phenolic compounds, due to the activation of plant defense mechanisms in response to abiotic and biotic stresses [91]. Moreover, mineral fertilization plays a variable role depending on conditions and crop type: specific combinations of nitrogen, phosphorus, and potassium can enhance total polyphenol content [92], while moderate application of fertilizers rich in macro- and micronutrients promotes the accumulation of flavonols and ellagic acid [93]. In contrast, excessive fertilization may negatively affect certain compounds, particularly anthocyanins [94].

Finally, sulfur fertilization has also been reported as an influencing factor on phenolic composition, leading to an increase in total polyphenols and phenolic acids under certain conditions [77]. These findings highlight the complexity of the effects of cultivation practices on phenolic compounds, which strongly depend on the interactions between agronomic and physiological factors.

Organic farming may promote higher polyphenol content as plants produce more phytochemicals as defense mechanisms [50]. Gentle harvesting methods that avoid damage help preserve polyphenol content. Post-harvest handling affects polyphenol profiles. Cold storage helps maintain polyphenol levels in fresh berries. MAP can extend shelf life while preserving phenolic compounds [95]. This variability explains wide ranges in reported polyphenol content in different studies (Table 2 and Table 3).

In Morocco, several studies have shown that the composition and polyphenol content of berries, vary considerably depending on environmental conditions, cultivation practices, and genetic factors. A study conducted by Sadik et al. [96] on seven samples of red and black raspberries sold in different markets in Morocco reported polyphenol contents ranging from 202 to 307.10 mg/100 g FW, this variability being attributed to differences in light intensity, wavelength, temperature, growing season, and geographic location. Moreover, climatic variability and water availability in Moroccan agricultural regions, particularly in irrigated areas such as the Gharb plain characterized by Mediterranean conditions and variable rainfall, can further influence crop growth and biochemical composition [96]. Similarly, Salim et al. [97], studying *Myrtus communis* from two distinct biogeographical zones, highlighted significant variability in extract yield and antioxidant compound content depending on environmental origin. High levels of phenolic compounds and flavonoids were associated with warmer, drier, and lower-altitude sites, emphasizing the combined effect of climatic and genetic factors.

Furthermore, studies conducted under Moroccan conditions have reported significant variability in phenolic compounds and antioxidant activity depending on genotype and interannual climatic conditions. Fruits grown in Morocco showed high concentrations of phenolic compounds, particularly total phenolic content (TPC) and total flavonoid content (TFC), accompanied by strong antioxidant activity, highlighting the influence of both genetic background and environmental factors on bioactive compound accumulation [98]. In addition, cultivation practices also play a crucial role, cultivation under organic systems has been associated with higher concentrations of beneficial phytochemicals in berries compared with conventional production [99]. According to Mounaimi et al. [100] higher polyphenol levels have been reported in organic blueberries, blackcurrants, raspberries, and strawberries compared to conventionally grown ones. Furthermore, organic farming experiences in Africa have shown that improving berry quality depends in particular on soil fertility management, biological pest control, biodiversity, market access, and training and support for farmers, promoting the adoption of sustainable agricultural practices [100].

The lack of extensive phytochemical profiling for Moroccan berries should not be viewed merely as a data gap, but as a strategic research frontier. Morocco’s unique agro climatic conditions characterized by high solar irradiance, significant UVB exposure, and controlled water deficit (precision irrigation) act as potent abiotic stressors. These environmental triggers are known to upregulate the phenylpropanoid pathway, specifically boosting the biosynthesis of anthocyanins and flavonols as a photoprotective mechanism [101,102]. Moroccan berries probably possess a unique “phenolic signature” compared to European counterparts, potentially featuring elevated levels of quercetin and myricetin glycosides synthesized to counteract oxidative stress induced by intense Mediterranean radiation. Empirical validation of this “terroir effect” is essential to define the functional superiority of Moroccan grown soft fruits.

## 4. Antioxidant Properties and Health Benefits

### 4.1. Mechanisms of Antioxidant Action

The antioxidant activity of raspberries, blackberries, and blueberries varies according to species and origin. According to several studies on Brazilian fruits, blackberries reached 77.8% 2,2-diphenyl-1-picrylhydrazyl (DPPH) inhibition at 100 µg/mL, slightly lower than blueberries (87.9%), while raspberries showed comparable values (red raspberry 87%, black raspberry 89%). For the 2,2′-azinobis-(3-ethylbenzothiazoline-6-sulfononic acid) (ABTS) assay, all three fruits exhibited moderate activity [54].

In contrast, studies on Moroccan blackberries revealed a very high antioxidant activity, with an IC50 as low as 0.1 mg/mL, directly associated with their richness in phenolic compounds (63.1 mg GAE/g DM), flavonoids (49.6 mg QE/g DM), and tannins (123.3 mg CE/g DM). This composition explains their strong radical-scavenging capacity, sometimes exceeding the Brazilian results, and confirms the direct correlation between phenolic content and antioxidant activity [103].

The antioxidant potential of berries is primarily governed by their polyphenolic composition, including anthocyanins, flavonols, flavanols, phenolic acids, and tannins [12,104]. This capacity varies across species and generally reflects differences in phenolic profiles, with blackberries exhibiting greater antioxidant activity than blueberries and raspberries [67]. In blueberries and raspberries, antioxidant capacity shows strong positive correlations with total phenolics (r = 0.83) and anthocyanins (r = 0.90), underscoring the central role of these compounds [105]. Collectively, these phenolic compounds confer protection against oxidative stress through multiple mechanisms, including free radical scavenging, metal chelation, and the modulation of antioxidant enzyme systems.

#### 4.1.1. Radical Scavenging and Metal Chelating Activity

**Figure 5:**
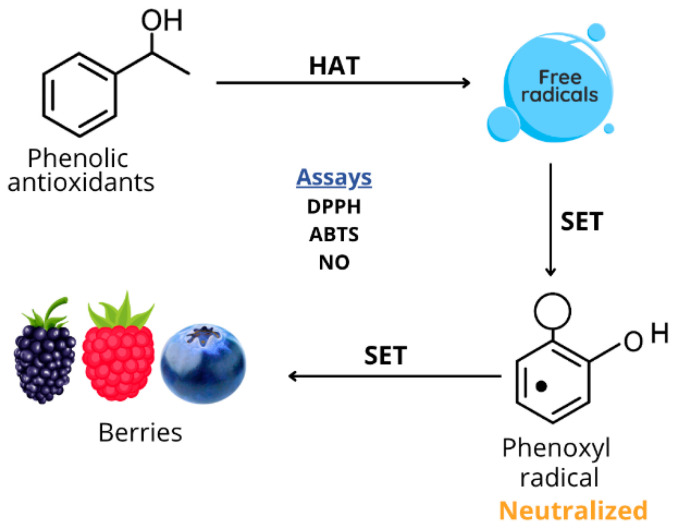
Mechanism of free radical scavenging by berry phenolic compounds.

Berry phenolic compounds act as potent free radical scavengers through electron donation and hydrogen atom mechanisms [106]. The hydroxyl groups on phenolic rings donate hydrogen atoms to reactive oxygen species (ROS) and reactive nitrogen species (RNS) (Figure 5), neutralizing them before they can cause cellular damage [107].

Anthocyanins are particularly effective antioxidants due to their multiple hydroxyl groups and conjugated double bond systems [108]. The antioxidant activity correlates with the number and position of hydroxyl groups on the B-ring [109].

**Figure 6:**
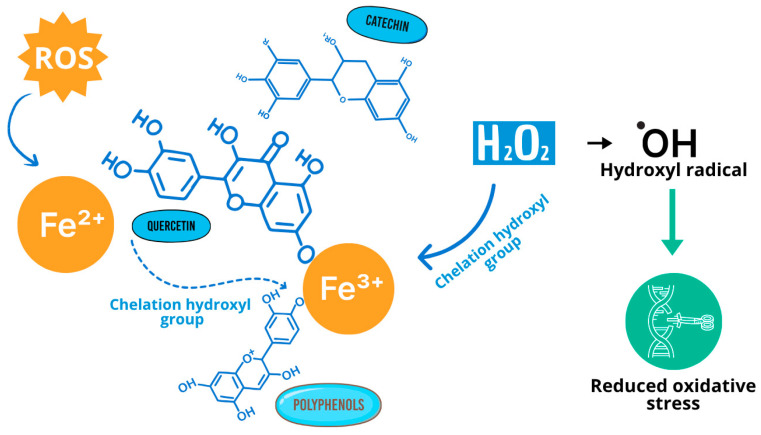
Schematic representation of metal chelation by berry-derived phenolic compounds.

Berry phenolic compounds can chelate transition metal ions such as iron and copper, preventing them from catalyzing Fenton reactions that generate hydroxyl radicals (Figure 6). This metal-binding capacity contributes significantly to the overall antioxidant properties of berries [110,111].

#### 4.1.2. Regulation of Antioxidant Enzymes

**Figure 7:**
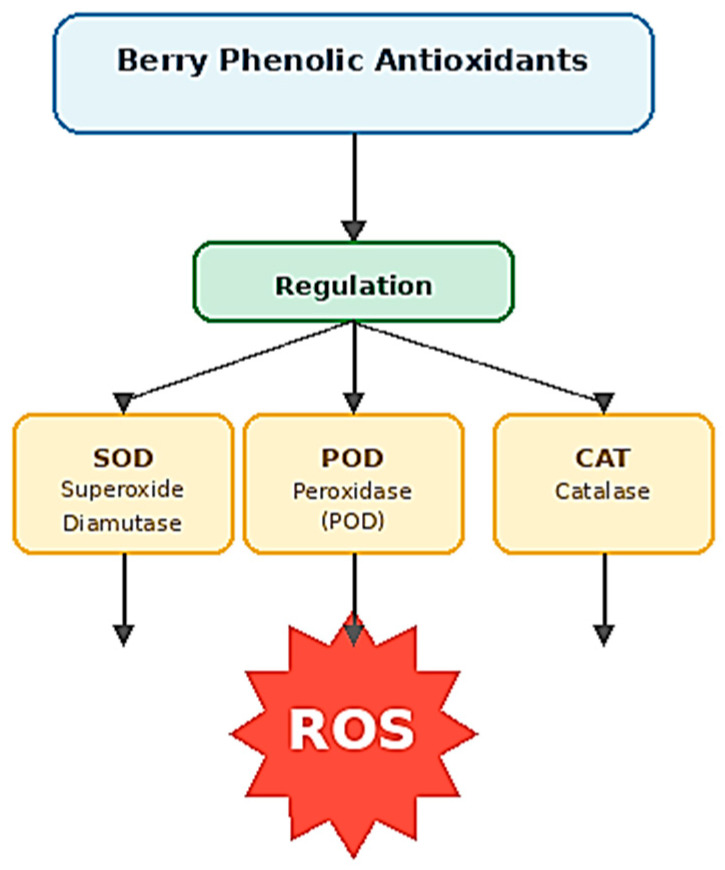
Mechanism of antioxidant enzyme regulation by berry phenolic compounds.

Beyond direct radical scavenging, berry phenolic compounds can modulate cellular antioxidant defense systems by upregulating endogenous antioxidant enzymes including superoxide dismutase (SOD), peroxidase (POD), and catalase (CAT) [112,113]. This indirect antioxidant mechanism provides sustained protection against oxidative stress (Figure 7).

### 4.2. In Vitro Assays: DPPH, ABTS, FRAP, ORAC, TPC

Multiple in vitro assays are used to evaluate berry antioxidant capacity, each measuring different aspects of antioxidant activity. The DPPH (2,2-diphenyl-1-picrylhydrazyl) assay measures free radical scavenging through electron transfer. The ABTS (2,2′-azino-bis(3-ethylbenzothiazoline-6-sulfonic acid)) assay evaluates both hydrophilic and lipophilic antioxidant capacity. The FRAP (Ferric Reducing Antioxidant Power) assay measures reducing potential. The ORAC (Oxygen Radical Absorbance Capacity) assay evaluates protection against peroxyl radicals. TPC is typically measured using the Folin-Ciocalteu method. Results from these assays generally correlate well with each other and with total polyphenol content, though no single assay fully captures the complex antioxidant mechanisms of berries [114,115].

### 4.3. Comparative Antioxidant Capacities of Blackberry, Raspberry and Blueberry

Comparative studies consistently show blackberries possess the highest antioxidant capacity, followed by raspberries and blueberries, correlating with their TPC patterns [84]. In DPPH assays, blackberries demonstrated IC50 values around 40.25 μM Trolox/L, compared to 21.78 μM Trolox/L for blueberries [116], and 1.5–3.4 mg/mL for raspberries [84]. FRAP values showed blackberries exhibiting 99.18 μM ferrous sulfate/L extract, blueberries 101.26 μM ferrous sulfate/L extract [116], and raspberries11.03 mg Fe^2+^/g DW [117]. However, specific cultivars and growing conditions can create significant variation. Some blueberry cultivars with exceptionally high anthocyanin content have shown antioxidant capacity exceeding typical blackberry values [116]. In order to obtain a comprehensive overview of the antioxidant potential of the berry extracts investigated, three complementary in vitro tests were assessed: the DPPH test, the ABTS test, and the FRAP test. The results of these tests are summarised in Table 4.

### 4.4. Impact of Drying, Freezing, and Processing on Antioxidant Properties

Processing methods have a considerable impact on the antioxidant properties of berries. Freezing generally preserves anthocyanin content well. Research conducted on blueberries has shown that there was no significant decrease in total anthocyanin content when samples were quick-frozen and stored at −20 °C for up to three months, compared to fresh samples, while drying resulted in losses of 41–49% [118]. Quick freezing minimizes ice crystal formation and cellular damage [119].

Drying methods show variable effects. Freeze-drying best preserves antioxidant compounds, while conventional hot-air drying can cause losses due to thermal degradation [120,121]. Spray drying enables intermediate retention of phenolic compounds relative to freeze drying, but results in significant losses of anthocyanins due to exposure to high temperature air intake. In blueberry juice, spray drying resulted in total phenolic compound losses averaging 76–78%, while anthocyanin losses reached approximately 57%; freeze-dried powders retained anthocyanin levels approximately 1.5 times higher than their spray-dried equivalent [122]. Thermal processing during jam production typically reduces total anthocyanin content by 27%, depending on temperature and duration, the rate of loss may increase, as the product is stored [123].

### 4.5. Health Benefits of Blackberry, Raspberry and Blueberry

**Figure 8:**
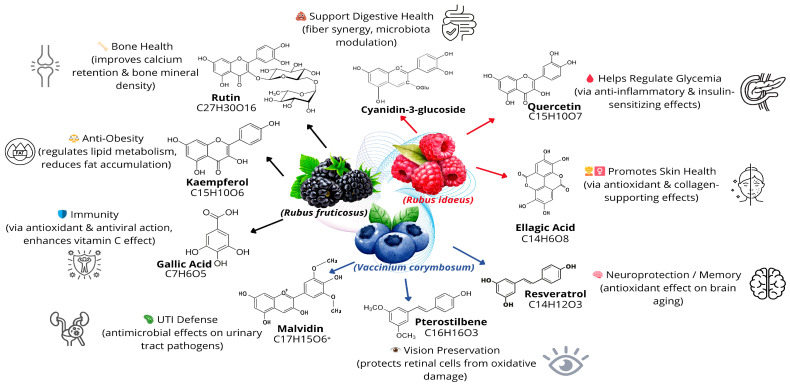
Health benefits of berry bioactive compounds across major physiological systems (created by A. Alahyane).

Interest in nutrition has greatly increased, not only for its role in providing energy but also for the prevention and management of diseases associated with insufficient, excessive, or unbalanced diets (Figure 8). [124]. Dietary habits vary across regions, but a balanced diet should provide adequate amounts of proteins, lipids, and essential micronutrients [125]. Diets based on red fruits, rich in vitamins and bioactive compounds, help reduce the risk of chronic diseases and can enhance the nutritional value of other foods when incorporated into the diet [3,126,127,128].

In contrast, Moroccan nutrition traditionally relies on a diet high in cereal products, particularly white flour and bread, which form the basis of many meals. This predominance of refined carbohydrates, often low in fiber and micronutrients, results in a high but nutritionally unbalanced caloric intake. Excessive consumption of these products, combined with low dietary diversity, promotes metabolic imbalances such as obesity, hypertension, and type 2 diabetes [129]. Moreover, this diet is very low in bioactive molecules capable of protecting the body against various diseases, including inflammation, certain cancers, obesity, and diabetes [117,130,131,132,133].

At this stage, it is necessary to incorporate red fruits into the Moroccan diet, in the form of various food products, to benefit from their richness in bioactive compounds. The high polyphenol content of berries translates into multiple health benefits, as demonstrated by in vitro, in vivo studies, and clinical trials.

#### 4.5.1. Anti-Inflammatory Effects

There are a number of mechanisms through which berry consumption demonstrates significant anti-inflammatory activity. Pro-inflammatory enzymes such as cyclooxygenase (COX) and lipoxygenase (LOX) are inhibited by phenolic compounds, which reduces the production of inflammatory mediators like prostaglandins and leukotrienes. Dietary phenolic compounds, including berry-derived flavonoids such as anthocyanins, achieve this through direct enzyme suppression and downregulation of NF-κB-mediated inflammatory signalling [134]. Research has shown that eating berries can reduce important markers of inflammation. However, the effect of berries on the body can vary depending on the type of berry, the amount eaten, and the person’s general health. In a randomised controlled trial, researchers found that consuming blueberries over 8 weeks in adults with metabolic syndrome produced measurable changes in oxidative stress and lipid profiles, though they did not find statistically significant CRP reductions across all participants. This suggests that anti-inflammatory responses to blueberry intake may be more consistently reflected in vascular and lipid endpoints than in acute-phase proteins [135]. A review of the research carried out to date on the subject found that consumption of raspberry has anti-inflammatory effects [136]. This is based on a meta-analysis of randomised controlled trials which found that TNF-α concentrations were significantly lower in the raspberry intake groups than in the control groups. For black raspberry specifically, supplementation significantly decreased inflammatory markers and improved the oxidative index compared to the placebo [136]. The protective effects of berries against chronic diseases, including cardiovascular disease, diabetes and neurodegenerative conditions, may be attributed to their anti-inflammatory properties.

#### 4.5.2. Cardiovascular Protection

Berry consumption provides significant cardiovascular protection through multiple pathways, including improvement of endothelial function, reduction of blood pressure, modulation of lipid profiles, and inhibition of platelet aggregation. Meta-analyses of randomized controlled trials demonstrate that regular berry consumption significantly improves cardiovascular risk factors. Pooled analysis across 22 RCTs encompassing 1251 subjects found that berry consumption significantly lowered LDL cholesterol (−0.21 mmol/L), systolic blood pressure (−2.72 mmHg), fasting glucose, and BMI (Body Mass Index) compared to control groups [137].

In a 6-month double-blind randomized controlled trial in adults with metabolic syndrome, daily consumption of one cup of blueberries significantly improved endothelial function, measured as a +1.45% increase in flow-mediated dilation, and reduced systemic arterial stiffness, though blood pressure and insulin resistance were not significantly affected [138]. These endothelial findings are corroborated by a separate systematic review and meta-analysis, which found blueberry consumption improved flow-mediated dilation by 1.50% across 11 pooled RCTs [139]. With regard to lipid profiles, a comprehensive meta-analysis of 44 RCTs confirmed that purified anthocyanin supplementation significantly reduced LDL cholesterol and triglycerides while increasing HDL cholesterol, with anthocyanin-rich berry consumption also significantly lowering total cholesterol and CRP [140].

Anthocyanins and other berry phenolic compounds also exhibit anti-thrombotic effects by inhibiting platelet aggregation and adhesion, potentially reducing the risk of thrombotic events. A randomized double-blind placebo-controlled crossover trial found that 320 mg/day of anthocyanin supplementation for 28 days reduced ADP-induced whole blood platelet aggregation by 29%, reduced monocyte-platelet aggregate formation by 39%, and inhibited platelet adhesion molecule expression, demonstrating a mechanism comparable to anti-platelet drugs [141]. These effects appear dose-dependent, with a subsequent RCT in dyslipidemic subjects confirming that anthocyanin supplementation inhibited collagen- and ADP-induced platelet aggregation in a dose-response manner, with effects becoming significant at doses of 80 mg/day or more over 12 weeks [142]. The cardiovascular benefits of berry consumption thus appear to operate through complementary vascular, lipid, and anti-thrombotic mechanisms, with regular long-term consumption showing greater effects than sporadic intake.

#### 4.5.3. Neuroprotection and Cognitive Function

Berries demonstrate neuroprotective effects relevant to cognitive health and neurodegenerative disease prevention. Berry-derived phenolic compounds and their colonic metabolites can cross the blood-brain barrier and exert direct neuroprotective actions in brain tissue. In vitro transport studies across blood-brain barrier endothelial cells at circulating concentrations have confirmed differential transport of berry phenolic sulfates, with evidence that these metabolites improve cellular responses to oxidative, excitotoxic, and inflammatory injuries, effects achieved in part through modulation of neuroinflammatory signaling pathways. Complementary animal evidence confirms that anthocyanins and their glucuronides from blueberry-fed pigs were detected in the cortex, cerebellum, and midbrain, establishing that berry anthocyanins and their metabolites do reach brain regions associated with learning and memory SELP [143].

Clinical trials demonstrate that berry consumption can meaningfully improve cognitive performance. In a preliminary randomized controlled trial, daily consumption of wild blueberry juice for 12 weeks in older adults with early memory changes resulted in significantly improved paired associate learning (*p* = 0.009) and word list recall (*p* = 0.04), with additional trends toward reduced depressive symptoms and lower glucose levels [144]. These findings have been replicated and extended in subsequent RCTs. A 12-week randomized, double-blind, placebo-controlled trial in middle-aged adults with subjective cognitive decline and insulin resistance found that daily blueberry powder supplementation improved performances on measures of lexical access (*p* = 0.003) and memory interference (*p* = 0.04), with participants also reporting reduced memory encoding difficulty in daily life activities [145].

The proposed mechanisms underlying these neuroprotective effects are multiple and complementary. Berry-derived phenolic compounds modulate brain resilience and function by enhancing synaptic plasticity and elevating hippocampal BDNF levels, with animal models of blueberry supplementation demonstrating potentiated cognition alongside elevated BDNF expression [146]. In a randomized, double-blind, placebo-controlled clinical trial in humans, polyphenol-rich berry and fruit supplementation significantly elevated plasma BDNF and CREB levels compared to placebo, with the authors concluding that these improvements occurred primarily through the reduction of oxidative stress and the regulation of signaling pathways associated with synaptic plasticity [147]. Additional mechanisms include reduction of neuroinflammation via suppression of NF-κB-mediated microglial activation, inhibition of amyloid-beta aggregation, and enhancement of cerebrovascular blood flow [148].

Long-term observational studies provide strong epidemiological support for the protective effects of berry consumption on cognitive aging. A prospective cohort of over 16,000 women followed for more than a decade, greater intakes of blueberries and strawberries were associated with significantly slower rates of cognitive decline, with the magnitude of the effect equivalent to approximately 1.5 to 2.5 years of delayed cognitive aging [149]. While these observational findings are highly consistent with clinical trial data, more large-scale, long-term randomized controlled trials are needed to establish definitive causal relationships between regular berry consumption and dementia risk reduction.

#### 4.5.4. Anti-Cancer Potential

Berries exhibit promising anti-cancer properties through multiple mechanisms, including inhibition of cell proliferation, induction of apoptosis, anti-angiogenic effects, and modulation of cell signaling pathways. Berry bioactives regulate carcinogen and xenobiotic metabolizing enzymes, various transcription and growth factors, inflammatory cytokines, and subcellular signaling pathways of cancer cell proliferation, apoptosis, and tumor angiogenesis, establishing a mechanistic basis for their chemopreventive potential [150].

In vitro studies demonstrate that berry extracts and isolated phenolic compounds inhibit the growth of various cancer cell lines. Extracts of six commonly consumed berries, blackberry, black raspberry, blueberry, cranberry, red raspberry, and strawberry, were evaluated against human oral, breast, colon, and prostate tumor cell lines. With increasing concentration of berry extract, increasing inhibition of cell proliferation in all of the cell lines was observed, with different degrees of potency between cell lines [151]. Black raspberries have attracted special interest due to their rich and diverse phytochemical composition. Black raspberries possess anti-proliferative properties, anti-inflammatory activity, activation of pro-cell-death pathways, modulation of the immune response, microbiome modulation, and reduction of oxidative stress [152]. In human cervical cancer cell lines, non-toxic levels of black raspberry extract significantly inhibited cell growth in a dose-dependent and time-dependent manner up to a maximum of 54–67%, while flow cytometry demonstrated induction of apoptosis in all treated cell lines [153]. Blackberries contain high levels of cyanidin-3-O-glucoside, a compound with prominent anticancer activity. Cyanidin-3-O-glucoside, isolated from blackberry, exhibited chemopreventive and chemotherapeutic activity, inhibiting UVB- and TPA-induced transactivation of NF-κB and AP-1, and reducing expression of cyclooxygenase-2 and tumor necrosis factor-α through inhibition of MAPK signaling activity [154]. While preclinical evidence is promising, large-scale human intervention trials are needed to confirm anti-cancer benefits and establish effective doses for cancer prevention or adjuvant therapy. Common themes across clinical studies support that black raspberries are anti-proliferative, anti-inflammatory, reduce oxidative stress, and restore tumor suppressive activity, though the precise dose, duration, and optimum mode of delivery for cancer inhibition remains to be fully elucidated [155].

#### 4.5.5. Gut Microbiota Modulation

Berry consumption significantly modulates gut microbiota composition and function, contributing to a wide range of downstream health benefits. The phenolic compounds content of berries, including ellagitannins and flavonoids such as anthocyanins, directly influences gut microbial populations, as these compounds reach the colon largely intact where they are metabolized by intestinal bacteria into smaller, more bioavailable phenolic compounds. The main site of metabolization of complex berry phenolic compounds is the gut through microbial action, and reciprocally, phenolic compounds and their metabolites modulate the microbial populations themselves [156].

Berry consumption consistently increases the relative abundance of beneficial bacterial populations, generally leading to higher levels of *Bifidobacterium*, *Lactobacillus*, and *Akkermansia*, suggesting a prebiotic-like effect [156]. Fermentation of berry anthocyanins also contributes to the generation of short-chain fatty acids, predominantly acetate, propionate, and butyrate, which protect gut barrier function, regulate pH, and inhibit invasion of enteric pathogens [157]. Specifically, blueberry and blackberry anthocyanin supplementation has been shown to restore SCFA levels and gut microbial balance disrupted by high-fat diet conditions, suggesting a role in ameliorating metabolic syndrome [158].

Different berry species show distinct microbiota-modulating profiles. Blueberry anthocyanin-rich extracts stimulate both probiotic bacteria (*Bifidobacterium*, *Lactobacillus*) and SCFA-producing bacteria (Roseburia, Faecalibaculum, Parabacteroides) [159]. Black raspberries demonstrate microbiome modulation that works synergistically with their anti-inflammatory and anti-cancer effects [152]. Raspberries, rich in ellagitannins and ellagic acid, produce notably different SCFA (Short-Chain Fatty Acids) profiles compared to other berries, reflecting the influence of specific polyphenol class composition on microbiome outcomes [160].

The relationship between berry phenolic compounds and gut microbiota is bidirectional. Berry compounds alter microbial populations while gut microbiota simultaneously transforms phenolic compounds into metabolites with distinct biological activities including anti-inflammatory and antitumor properties. The production of these beneficial metabolites is strongly dependent on the individual’s gut microbial composition, which explains in part the interindividual differences in health outcomes observed after polyphenol intake [161].

### 4.6. Antidiabetic and Anti-Obesity Effects

Berry fruits offer substantial antidiabetic and anti-obesity benefits through their bioactive compounds. Polyphenolic compounds, especially anthocyanins, exert potent antioxidant and anti-inflammatory properties that may help prevent obesity-related disorders including type 2 diabetes by targeting multiple metabolic pathways simultaneously [162].

In more recent work, blueberry and blackberry anthocyanin supplementation restored disrupted SCFA levels and gut microbial balance caused by high-fat feeding, suggesting an indirect metabolic benefit through microbiota modulation in addition to direct anti-obesity effects [158].

The primary mechanisms underlying these antidiabetic effects include inhibition of carbohydrate-metabolizing enzymes crucial for glucose regulation. Both anthocyanins and acarbose target α-glucosidase and pancreatic α-amylase breakdown, reducing the amount of glucose released into the bloodstream, with anthocyanin-rich berry extracts showing inhibitory effects many times higher than those elicited by acarbose in some preparations [163]. In human intervention studies, a meta-analysis of randomized controlled trials found that dietary anthocyanin intake at a median dose of 320 mg/day significantly reduced fasting blood sugar (−2.70 mg/dL), 2-h postprandial glucose, HbA1c, and HOMA-IR compared to control groups, with effects being most consistent in participants with type 2 diabetes [164]. These glycemic improvements were rated as high-quality evidence by GRADE assessment for FBG and HbA1c outcomes [165].

The anti-obesity effects of berries are further enhanced by their capacity to modulate gut microbiota composition, increase production of beneficial short-chain fatty acids, and reduce systemic inflammation, mechanisms that collectively contribute to improved insulin sensitivity and metabolic homeostasis [158]. The unique combination of bioactive compounds present in berries thus offers a multifaceted approach to the prevention and management of metabolic disease.

**Table 4:** Biological activities of berries.

Biological Activity	Assay Method	Berry Type/Variety	Extract Details	Quantitative Results	References
Antioxidant	DPPH	Blackberry (‘TripleCrown’)	Methanol/Water (80:20)	IC50 = 184.43 µg/mL	[117]
	DPPH	Blackberry	methanol	IC50 97.1 ± 2.4 µg DW/mL	[166]
	FRAP	Blackberry (‘TripleCrown’)	Methanol/Water (80:20)	17.28 mg Fe^2+^/g DW	[117]
	FRAP	Raspberry (‘Encore’)	Methanol/Water (80:20)	11.03 mg Fe^2+^/g DW	[117]
	DPPH	Raspberry (‘Encore’)	Methanol/Water (80:20)	IC50 = 316.02 µg/mL	[117]
	ABTS	R. idaeus/R. occidentalis Hybrid (R1314701)	Lyophilized/Methanol	2.31 mmol TE/g DW	[167]
	FRAP	R. occidentalis ‘Jewel’ ‘Bristol’ A Bristol’ B MacBlack Niwot Heban	Lyophilized/Methanol	3.47–4.09 mmol TE/g DW	[167]
Antimicrobial	Zone of Inhibition (*S. aureus*)	Blackberry (*R. weberbaueri*)	Hydromethanolic	14 ± 1.26 mm	[168]
	Zone of Inhibition (*E. coli*)	Blackberry (*R. andicola*)	Hydromethanolic	11.5 mm	[168]
	MIC (*S. aureus*)	wild blackberry	GW-glycerol-water	7.4 mm	[169]
	MIC (*Enterococcus faecalis*)	wild blackberry	GW-glycerol-water	9.5 mm	[169]
	Inhibition *S. aureus*	Blackberry (Fruit)	GW-glycerol-water	13.95 ± 0.63 mm	[170]
	Inhibition *L. monocytogenes*	Blackberry (Fruit)		19.20 ± 0.87	[170]
Anti-inflammatory	Cytokines IL-8 produced by HT-29 and T-84	Blackberry	Phenolic Extract	1.56 mg/mL	[166]
	Signaling Pathways	Raspberry (*R. idaeus*)	Polyphenols	MAPK & NF-κB Inhibition	[130]
	Signaling Pathways	Blueberry	Anthocyanins	NF-κB Inhibition	[171]
Anticancer	E2-induced mammary cancer	Blueberry	powdered berries	1% or 2.5% powdered berries (BR or BB) or 400 ppm EA	[172]
	Human CRC cell lines	Blackberry	Blackberry crude extract	8–96 mL/mL which is equivalent to 167.4–277.1 mg/mL total phenolic or 47.1–78 mg/mL total monomeric anthocyanin contents	[131]
	Clonogenic survival assay and caspase-3 activity kits	Raspberry	Raspberry extract (RE)	50 μg/mL of RE	[173]
Antidiabetic	α-Glucosidase	Blueberry	Anthocyanins extract	IC50 = from 68.33 to 218.2 μM	[132]
	α-amylase and α-glucosidase inhibitory activities	Blackberry (*Rubus* spp.)	Purified anthocyanin extracts	IC50 = 0.10 and 0.06, 0.56 and 0.32, and 3.98 and 2.16 mg/mL	[174]
	α-Glucosidase	Raspberry	Raspberry exracts	IC50 = 16.8 to 34.2 µg/mL	[175]
Cardioprotective	LDL Oxidation	Wild Blueberry juice	Bluebeery polyphenols	44.5 ± 1.7 (U/L)	[176]
	LDL Oxidation HDL Oxidation PPAR-α receptor subtype	raspberry	raspberry ketone	doses of 100 and 200 mg/kg	[133]
	Thiobarbituric acid reactive substances (TBARS) assay	Blackberry	Blackberry MeOH extract	IC50 = 2.51 ppm	[177]
Neuroprotective	Lowering the gene expression of MusIL-1b, MusNLRP3, MusCaspase-1, MusASC, MusTNF-a, MusIL-6, and MusiNOS and the protein expression of NLRP3 and Caspase-1	Blueberry	BB extracts	significantly inhibit the gene expression	[178]
	BV-2 microglia cell viability (CTG 2.0 cell viability assay)	Blackberry	Anthocyanins extracts	80 µg/mL	[179]
	BV-2 microglia cell viability (CTG 2.0 cell viability assay)	Red Raspberry	Anthocyanins exctracts	80 µg/mL	[179]

### 4.7. Moroccan Dietary Pattern and Health Context

Morocco’s traditional dietary pattern closely aligns with the Mediterranean diet model, characterized by olive oil as the principal fat source, whole grains, legumes, fruits, vegetables, and a rich tradition of herbs and spices including cumin, turmeric, cinnamon, and ginger [180]. However, berry consumption remains relatively low compared to other Mediterranean regions, with traditional fruit consumption centered primarily on citrus fruits, dates, figs, and pomegranates. The national STEPS survey conducted in 2017–2018 revealed that 76.3% of Moroccan adults did not meet the recommended daily intake of fruits and vegetables [181], highlighting a significant gap in dietary phytochemical intake at the population level.

Morocco is currently undergoing a rapid nutrition transition, shifting away from its traditional dietary heritage toward a Western dietary pattern heavy in sugar, and fat, driven by urbanization, economic growth, and changing food environments [180]. The same national STEPS survey found that 53% of Moroccan adults were overweight and 20% were obese, 29.3% were hypertensive, and 10.6% had diabetes, with an additional 10.4% classified as pre-diabetic. The cardiovascular disease burden is particularly alarming: in Morocco, 80% of total deaths are due to non-communicable diseases, with cardiovascular disease as the primary cause of death at 38%, followed by cancer at 18%. Between 2007 and 2017, diabetes mortality progressed from the 9th to the 4th position as a leading cause of death, representing an increase of 35.4% [182].

These epidemiological trends indicate an urgent need for evidence-based preventive dietary interventions. Berries, with their well-documented anti-inflammatory, antioxidant, antidiabetic, and cardiovascular-protective properties, represent a promising and culturally compatible addition to Moroccan preventive health programs, directly targeting the metabolic pathways underlying the country’s growing non-communicable disease burden.

## 5. Bioavailabilty and Consumption Recommandations

**Figure 9:**
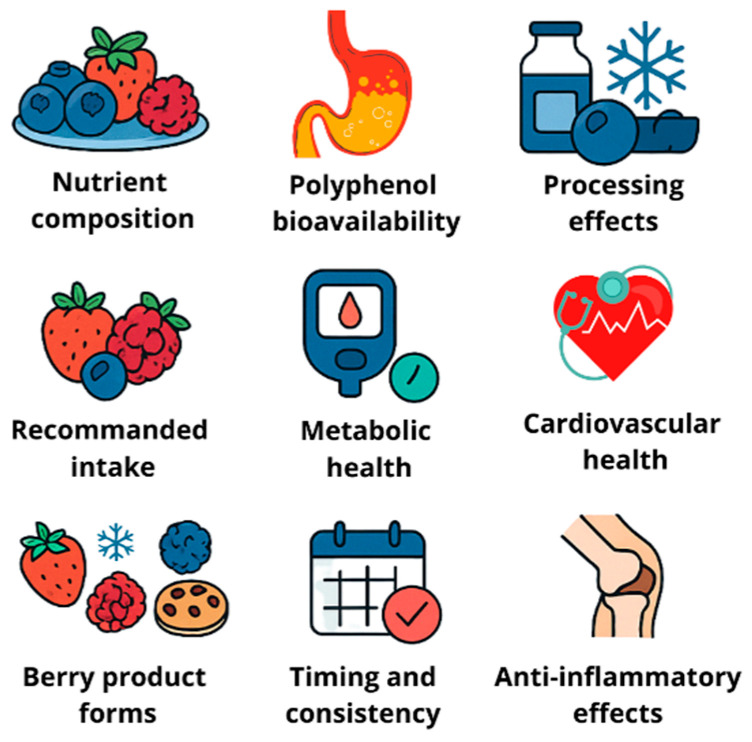
Bioavailability and consumption guidelines for berries: nutritional, metabolic, and cardiovascular perspectives.

As illustrated in Figure 9, berry consumption recommendations are based on multiple interconnected factors, including:

**Nutrient composition:** Berries are low in calories while providing essential vitamins (particularly vitamin C), minerals, and dietary fiber, making them excellent foods for weight management and overall health [183].

**Polyphenol bioavailability:** Berry phenolic compounds, especially anthocyanins, are absorbed intact at very low levels (<1% of intake), yet they appear to exert systemic effects [183,184]. This paradox can be resolved by recognizing that their metabolites (e.g., phenolic acids, conjugated forms produced by the gut microbiota) reach much higher concentrations in the circulation and may be the true bioactive species. Few studies have simultaneously measured parent compounds, microbial metabolites, and clinical outcomes in humans. Understanding inter individual variability in polyphenol metabolism (driven by genetics and baseline microbiota) remains a major frontier that could ultimately enable personalized dietary recommendations.

**Processing effects:** Different processing methods (freezing, drying, juicing) affect polyphenol content and bioavailability differently. Freezing preserves most phenolic compounds, while thermal processing can cause significant losses.

Drying processes play a crucial role in preserving the bioactive compounds of berries. A comparative study on raspberry drying using hot air and freeze-drying showed that freeze-drying better preserves total phenolic compounds (82%) and anthocyanins (89%), whereas hot air-drying leads to significant losses, reaching 63% and 55%, respectively [185].

Similarly, a study conducted on blueberries, comparing hot air drying (60 and 90 °C) with freeze-drying, revealed that freeze-dried berries retain high levels of total phenolic compounds (TPC) and total flavonoids (TFC). In contrast, hot air drying has a greater negative impact on these compounds, particularly at 90 °C [58].

Furthermore, another study on blackberries demonstrated that quick-freezing (−34 °C for 25 min) is more effective than slow freezing (−18 °C for 24 h) in preserving vitamin C, total polyphenols, anthocyanins, and antioxidant capacity, while also minimizing color degradation [186].

**Recommended daily intake:** Consuming 1–2 portions of berries daily, equivalent to approximately 150–300 g fresh berries or 75–150 g frozen berries, appears to provide significant health benefits based on intervention studies [187].

**Metabolic health benefits:** For individuals with insulin resistance or type 2 diabetes, regular berry consumption (150–300 g daily) has shown improvements in glycemic control and insulin sensitivity [187].

**Cardiovascular health dosage:** Meta-analyses have shown that berry consumption can significantly lower LDL-cholesterol (−0.21 mmol/L), systolic blood pressure (−2.72 mmHg), fasting glucose (−0.10 mmol/L), body mass index (−0.36 kg/m^2^), HbA1c (−0.20%), and inflammatory markers like TNF-α (−0.99 ρg/mL) [137].

**Berry product forms:** Berry benefits can be obtained from various forms, including fresh, frozen, dried, and processed products. A four-week intervention using cookies containing blueberry and raspberry pomace showed significant reductions in LDL-cholesterol (20.16%) and increases in adiponectin levels (25.52%) in healthy female subjects [188].

**Timing and consistency:** Regular, consistent consumption appears more beneficial than occasional intake. Long-term daily consumption is associated with sustained health benefits across multiple systems [187].

**Synergistic effects:** Berries may be most effective when consumed as part of a balanced diet rather than as isolated supplements. Their phytonutrients work synergistically with other dietary components to reduce the risk of chronic diseases [189,190].

**Special population considerations:** Different populations may benefit from specific amounts; older adults may need higher intakes for cognitive benefits, while those with metabolic syndrome may benefit from regular moderate amounts [190].

## 6. Challenges and Research Gaps

While the evidence for berry health benefits is substantial and consistent across many studies, it is essential to acknowledge the limitations: variability in composition, reliance on surrogate endpoints, lack of long term hard endpoint trials, incomplete understanding of mechanisms, and the prevailing reductionist approach that overlooks matrix effects and nutritional dark matter. Future research should prioritize standardized preparations, rigorous bioavailability assessments, whole matrix intervention designs, and large scale, long term RCTs that integrate microbiota and metabolomic analyses.

### 6.1. Variability in Phenolic Compounds Content

One of the primary challenges in berry research is the substantial variability in polyphenol content across cultivars, growing conditions, harvest timing, and post-harvest handling. This variability complicates dose-response relationships and makes it difficult to establish standardized recommendations. For example, TPC in blueberries can range from 48 to 304 mg GAE/100 g of fresh weight depending on these factors [66].

### 6.2. Processing and Preservation Losses

Processing methods significantly affect polyphenol retention in berries. Freezing at −18 °C is widely recognized as the most suitable strategy for maintaining both nutritional value and color quality in berry products, with studies reporting total phenolic retention of 79–87% across blackberry, raspberry, blueberry, and sour cherry pulps after 12 months of frozen storage [191]. In contrast, thermal processing causes significant polyphenol losses: pasteurization of blackberry juice at 75 °C for 15 s and 92 °C for 10 s reduced antioxidant scavenging capacity by 26–27% compared to non-pasteurized juice, while anthocyanin concentrations decreased significantly in both conditions [192].

### 6.3. Standarization of Analytical Protocols

The lack of standardized analytical protocols for polyphenol quantification across laboratories leads to inconsistent results and difficulties comparing studies. The Folin-Ciocalteu method, the most widely used for total polyphenol quantification, is carried out using widely varying experimental factors, including reagent concentrations, absorbance systems, and reference standards, making published results broadly variable and difficult to compare [193]. Extraction protocols introduce further variability, with enzymatic versus conventional methanolic extraction alone producing differences of over 25% in reported polyphenol values [194]. For individual compound identification, LC-MS/MS is the most powerful technique, yet different extraction and chromatographic conditions across laboratories make inter-laboratory comparison difficult even on the same analytical platform [195]. International harmonization of analytical methods, including standardized extraction solvents, unified reference standards, and validated quantification platforms, would substantially improve the comparability and reliability of polyphenol data across studies.

### 6.4. Lack of Clinical Studies and Research Limitations

Most evidence supporting berry health benefits originates from in vitro studies and animal models, which provide valuable mechanistic insights but do not reliably translate to human health outcomes. Large-scale, long-term, placebo-controlled human trials remain scarce, and existing studies are further limited by small sample sizes, short intervention periods, and significant methodological heterogeneity in berry form, dose, and population characteristics, making cross-study comparisons and meta-analytic conclusions difficult [196]. Establishing definitive dose-response relationships, understanding individual variability in biological response due to genetics and gut microbiota composition, and characterizing the bioactivity of gut-derived polyphenol metabolites represent the most critical remaining research gaps [197]. Biomarker development also remains limited, as self-reported dietary questionnaires lack precision, and validated biomarker panels for berry intake have only recently begun to emerge [198]. Finally, the majority of berry health studies have been conducted in Western populations, leaving significant gaps in evidence for diverse dietary contexts including North African and Middle Eastern populations.

## 7. Future Research Directions

Despite promising evidence of berries’ health benefits, several critical research directions must be pursued to advance our understanding and practical applications.

**Clinical trials:** Large-scale, long-term randomized controlled trials are needed to establish causal relationships between berry consumption and health outcomes, particularly for cardiovascular disease, diabetes, cognitive decline, and cancer prevention. Future RCTs should use standardized berry preparations with characterized polyphenol content, assess bioavailability rigorously, and evaluate clinically meaningful endpoints rather than surrogate biomarkers alone [199].

**Bioavailability and metabolism:** More research is needed on polyphenol absorption, distribution, metabolism, and excretion (ADME), including the role of gut microbiota in generating bioactive metabolites. Plasma and urinary polyphenol metabolite profiles differ substantially across berry species and individuals, and controlled dietary intervention trials combining GC-MS and HPLC analysis are needed to fully characterize the bioavailability of phenolic compounds from specific berry sources [200]. Understanding inter-individual variation in polyphenol metabolism, driven by genetics, gut microbiota composition, and metabotype, could ultimately enable personalized nutrition recommendations [197].

**Mechanism elucidation:** While multiple mechanisms have been proposed for berry health benefits, definitive pathways remain unclear. Testing the hypothesis that accumulation of polyphenolic compounds above a threshold level, rather than the presence of specific individual polyphenols, is key to health protection will require targeted human clinical trials that go beyond simple dose-finding designs [201]. Research should clarify which specific compounds and mechanisms are most important for each distinct health outcome.

**Dose-response relationships:** Systematic studies establishing dose-response curves for various health endpoints are needed to enable evidence-based consumption recommendations. The limited number of published randomized clinical trials that measure both biological endpoints and microbiota changes simultaneously, only three identified in a comprehensive systematic review highlights the extent of this gap [202].

**Comparative effectiveness:** Direct comparisons of different berry types, cultivars, and forms, fresh, frozen, dried, and extract, would help identify optimal choices for specific health goals. Standardization of berry form, dose, and intervention duration across studies is a prerequisite for meaningful cross-study comparisons and reliable meta-analytic conclusions [196].

**Agricultural optimization:** Research on cultivation, harvesting, and post-harvest practices that maximize polyphenol content while maintaining sustainability and economic viability remains an important practical priority [203]. Additionally, the vast majority of berry health studies have been conducted in Western populations; expanded research in diverse ethnic and dietary contexts, including North African, Middle Eastern, and Asian populations, is essential to establish globally applicable recommendations.

## 8. Current Status and Future Perspectives of Berries as Functional Foods

Berries are increasingly recognized as functional foods due to their rich polyphenolic content and associated health benefits, with current evidence supporting their role in cardiovascular health, metabolic regulation, cognitive function, and reduction of chronic inflammation. The beneficial health effects of berries are derived from a multifactorial combination of phenolic compounds, antioxidants, and their metabolites acting synergistically with dietary fibers and vitamins [43]. Future development will focus on incorporation into fortified foods and beverages using whole berry purees and concentrated extracts, innovation in encapsulation and freeze-drying technologies to enhance polyphenol bioaccessibility and stability, development of standardized nutraceutical formulations, and integration of berry-derived ingredients into dietary guidelines and public health recommendations [204,205].

Morocco presents significant opportunities to develop its berry industry as both a domestic health food resource and an export commodity. Over 14,000 hectares of berries are currently cultivated nationwide, including blueberries, raspberries, and blackberries and Morocco has consolidated its position as the world’s fourth-largest berry exporter, leveraging favorable climatic conditions, geographical proximity to European markets, and competitive production capacity [206,207]. However, persistent challenges including water scarcity, climate volatility, rising input costs, and stricter European phytosanitary regulations require coordinated investment in sustainable irrigation, cultivar adaptation, and applied scientific research to ensure long-term industry viability and maximize the nutritional quality of Moroccan-grown berries for both export and local health programs.

## 9. Integration in National Health Strategies

As scientific evidence continues to accumulate on the health benefits of berries, there is growing potential for their formal integration into national dietary guidelines and public health strategies. Berries are increasingly recognized in dietary frameworks, the Nordic Nutrition Recommendations 2023 explicitly acknowledge the role of berries in preventing chronic diseases and supporting their inclusion in food-based dietary guidelines alongside fruits and vegetables [208]. In the United States, the 2025 Dietary Guidelines Advisory Committee process has examined the nutritional impact of incorporating berries as a distinct subcategory within the fruit group, given that a more refined subcategorization of fruit in dietary guidance may be scientifically justified based on their distinctive polyphenol and anthocyanin profiles [209]. Consumer interest in berries as health-promoting foods has similarly grown substantially, driven by heightened awareness of their nutritional value and the broader shift in food consumption patterns where health benefits are increasingly weighted alongside taste and convenience.

Formal integration of berries into national health strategies will nonetheless depend on further research through large-scale, long-term human intervention trials to establish clear causal relationships and evidence-based dose recommendations for specific health outcomes [210].

## 10. Innovation in Extraction and Valorization (Cosmetics, Nutraceuticals, Supplements)

Berry valorization now extends beyond fruits to utilize entire plants, including pomace, leaves, and seeds. Berry pomace contains substantial bioactive compounds and has been successfully incorporated into gluten-free cookies with “high fiber” nutrition claims [211].

Berry leaves contain higher phenolic levels and enhanced antioxidant properties compared to fruits, including phenolic acids, flavonols, anthocyanins, and procyanidins, creating opportunities for food additives and nutraceuticals [212]. Berry seeds contain phenolic compounds, fatty acids, and tocopherols with species-specific profiles suitable for oils, extracts, or flour in functional foods, pharmaceuticals, and cosmetics [213].

Ultrasound-assisted extraction effectively recovers anthocyanins from blueberry pomace while preserving bioactive compound integrity [214]. Berry extracts incorporated into chocolates increase polyphenol content and antioxidant capacity [215], while raspberry and blueberry byproducts improve antioxidant properties in food formulations [216].

Black raspberry extracts demonstrate anti-cancer properties in preclinical and clinical trials, with berry phytochemicals showing antioxidant, anti-inflammatory, anticancer, and antimicrobial properties [16,217]. Breeding programs are developing markers for enhanced nutritional properties, quality, and nutraceutical content [218].

Global raspberry production reached 840,000 tonnes in 2017, with raspberries dominating strawberries and blackberries in 2020 [219]. Valorization approaches extend commercial use beyond fresh markets despite berry perishability challenges [220].

To enhance the global competitiveness of the Moroccan berry sector, there is a clear need to transition from purely quantitative yields to qualitative, bioactive based standardization. The establishment of a Protected Geographical Indication (PGI) or a “Quality Label” based on specific phytochemical fingerprints (e.g., high cyanidin 3 O glucoside content or specific flavonol profiles) would provide a significant market advantage [221]. Such a framework would allow Morocco to market its berries not just as agricultural commodities, but as high potency functional foods, justifying premium pricing and ensuring long term sustainability in the international market.

## 11. Conclusions

Morocco ranks among the leading global exporters of berries, occupying the fourth position due to favorable agroclimatic conditions and a rapidly expanding production sector. This context offers promising opportunities, both economically and for the promotion of strategies aimed at preventing non-communicable diseases. However, the lack of detailed metabolomic profiling and cultivar-specific data still limits the optimal integration of these fruits into the development of functional foods. Nevertheless, the convergence between a growing export market and public health needs positions berries as strategic resources in combating chronic diseases, provided that research focuses on local varieties and their specific bioactive compounds.

In this context, the present review highlights that raspberries (*Rubus idaeus*), blackberries (*Rubus fruticosus*), and blueberries (*Vaccinium corymbosum*) cultivated in Morocco are rich sources of phytochemicals, particularly bioactive polyphenols. Berries from regions such as Gharb, Loukkos, and Souss-Massa are especially notable for their high anthocyanin content, with blackberries exhibiting the strongest antioxidant activity. These compounds exert beneficial effects through mechanisms such as free radical scavenging and modulation of inflammatory processes. Evidence from meta-analyses suggests that regular consumption contributes to the improvement of cardiovascular risk factors, including reductions in blood pressure and cholesterol levels, while also showing promising effects in neuroprotection, gut health, and anticancer activity.

However, several methodological limitations remain. Most available studies are short-term, which restricts the assessment of long-term effects. In addition, variability in polyphenol bioavailability, combined with interindividual differences in gut microbiota, complicates the interpretation of findings, especially since few studies simultaneously assess microbiota changes and clinical outcomes. Furthermore, disparities in analytical methods across laboratories, along with the predominance of clinical trials conducted in Western populations, limit the applicability of these findings to North African populations.

To enhance the valorization of Moroccan berries and align them with international health standards, several research priorities should be addressed. It is essential to develop bioavailability studies integrating in vitro digestion models and clinical trials to better understand polyphenol metabolism. The implementation of long-term randomized controlled trials (≥6 months), using standardized berry preparations, would allow for robust associations between individual responses and gut microbiota profiles. Moreover, it is crucial to evaluate the impact of local processing methods and agroclimatic conditions on anthocyanin stability and secondary metabolite production. Finally, conducting population-specific dose–response studies will support the development of tailored dietary recommendations. Furthermore, based on the progressive increase in red fruit production in Morocco, it is necessary to establish labels such as a “Protected and Controlled Designation of Origin” (AOC/AOP) to ensure traceability, quality, and protection of these local fruits, while enhancing their economic and nutritional value.

## Figures and Tables

**Table 1:** Botanical and agricultural characteristics of the three berries.

Species	Common Name	Family	Morphological Features	Growing Season	Cultivation Region in Morocco	Main Cultivated Varieties in Morocco
***Rubus idaeus***	Raspberry	Rosaceae	Shrub, red fruits, thorny stems	Spring–Summer	Gharb, Souss-Massa, larache	Cardinal, Sevillana, Carmina, Alicia, Maravilla, Marabia, Glen Lyon, Brilliance, Adelita (low-chill)
***Rubus*** ***fruticosus***	Blackberry	Rosaceae	Thorny vine, dark purple fruit	Late Summer	Gharb, Souss-Massa Loukkos (Larache)	Brazos, Rosborough, Tupi (upright habit); Stella Blue, Violeta (new varieties)
***Vaccinium*** ***corymbosum***	Blueberry	Ericaceae	Woody shrub, blue fruit	Spring–Summer	Gharb, Souss-Massa Loukkos (Larache) (under greenhouse trials)	Sharp Blue, Misty, Biloxi, Gulf Coast, Emerald, Jewel, Star, Sapphire, Blue Crisp, Millenia, Windsor (off-season bearing)

## Data Availability

No new data were created or analyzed in this study.
